# Two rare ophiocordycipitaceous fungi newly recorded in Taiwan

**DOI:** 10.1186/s40529-015-0110-x

**Published:** 2015-10-24

**Authors:** Yi-Hong Ke, Yu-Ming Ju

**Affiliations:** 1 Department of Life Science, National Taiwan University, Taipei, Taiwan; 2 Institute of Plant and Microbial Biology, Academia Sinica, Taipei, Taiwan

**Keywords:** Clavicipitaceae, Cordycipitaceae, *Elaphocordyceps*, Hypocreales, *Ophiocordyceps*, Ophiocordycipitaceae, *Tolypocladium*

## Abstract

**Background:**

Ophiocordycipitaceae is a highly diverse fungal family parasitizing a wide range of arthropods and hypogeous fungi. We collected two ophiocordycipitaceous species previously unknown in Taiwan: one emerged from hypogeous fruiting bodies of an *Elaphomyces* fungus and the other was associated with dragonflies.

**Results:**

Based on gross morphology, microscopic features, ITS sequences, and hosts, the two ophiocordycipitaceous fungi were identified as *Tolypocladium japonicum* and *Ophiocordyceps odonatae*. We isolated axenic cultures of these two fungi, and their anamorphs were obtained. The simplicillium-like anamorph of *T. japonicum* is described herein for the first time. The anamorph of *O. odonatae* produce conidia holoblastically in sympodial sequence and is assignable to *Hymenostilbe*. A dichotomous key to the species of Ophiocordycipitaceae reported in Taiwan is provided.

**Conclusion:**

A thorough literature study indicates that the two fungi reported herein have rarely been collected. Our identifications of *T*. *japonicum* and *O*. *odonatae* agree well with descriptions in the literature and are highly supported by DNA sequence analysis.

## Background

Ophiocordycipitaceous fungi are parasites of more than ten orders of Arthropoda and one fungal genus *Elaphomyces* (Kobayasi 1941, 1981, 1982; Kobayasi and Shimizu 1960; Mains 1957, 1958; Spatafora et al. 2007). Taxa of Ophiocordycipitaceae G. H. Sung et al. were included in Clavicipitaceae (Lindau) Earle ex Rogerson sensu lato primarily because they have fleshy stromata, filiform ascospores, and unitunicate asci with a cap-like thickening on top (Kobayasi 1941, 1981, 1982; Kobayasi and Shimizu 1960; Mains 1957, 1958). Molecular phylogenetic studies (Sung et al. 2007a, b; Spatafora et al. 2007) suggested that Clavicipitaceae sensu lato can be segregated into three families: Cordycipitaceae Kreisel ex G. H. Sung et al., Ophiocordycipitaceae, and Clavicipitaceae sensu stricto. Ophiocordycipitaceae differs from the Cordycipitaceae as circumscribed by Sung et al. (2007a) mainly in having dark, tough, fibrous to pliant stromata at maturity. In accordance with the changes in Art. 59 of ICN, Quandt et al. (2014) proposed that Ophiocordycipitaceae contains the following six genera: *Ophiocordyceps* Petch, *Purpureocillium* Luangsa-ard et al., *Tolypocladium* W. Gams, *Harposporium* Lohde, *Drechmeria* W. Gams & H.-B. Jansson and *Polycephalomyces* Kobayasi. However, many ophiocordycipitaceous fungi still await to be reassigned to a proper taxonomic position.

**Fig. 1:**
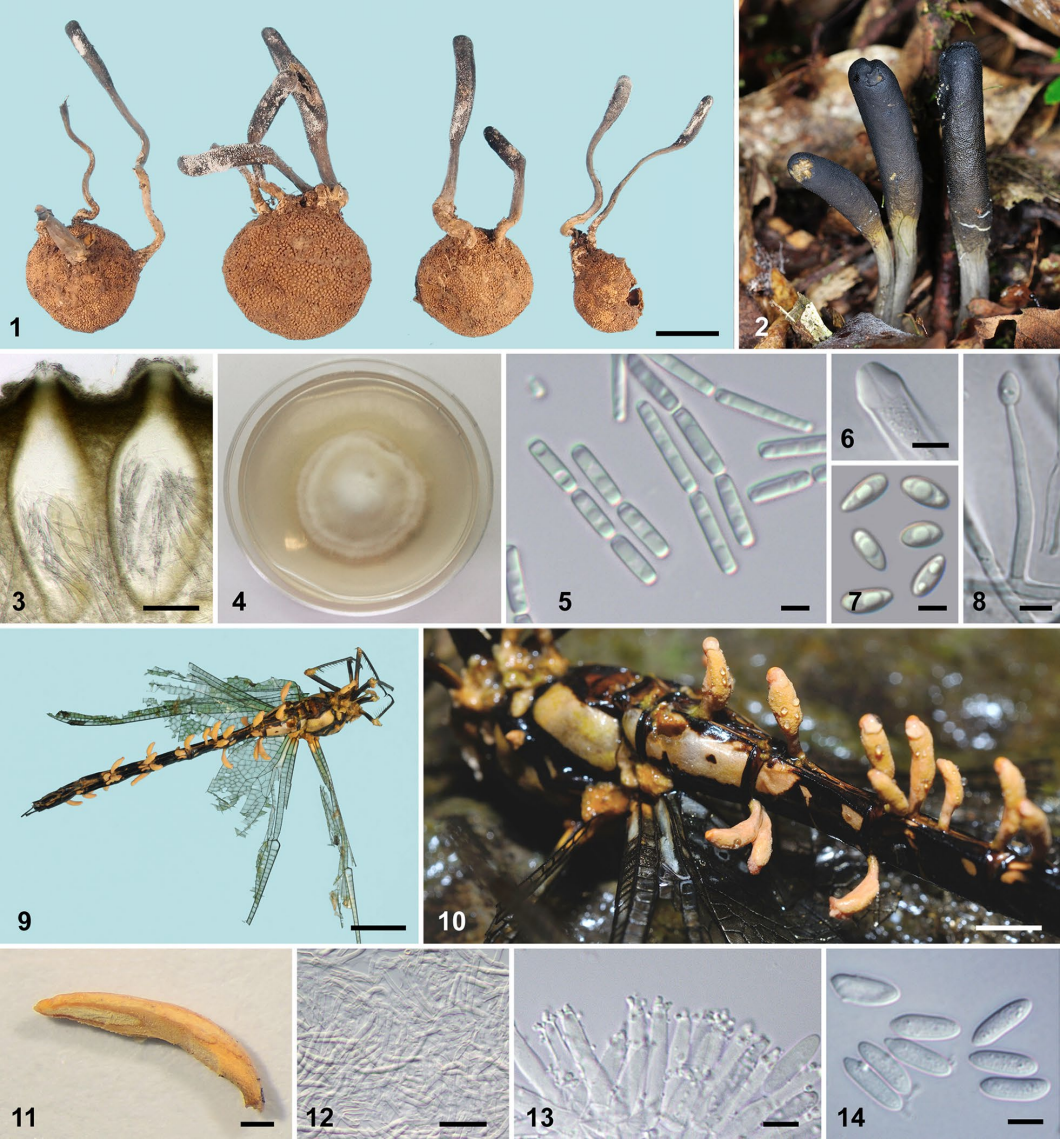
*Tolypocladium japonicum* and *Ophiocordyceps odonatae*. Figure **1**–**8** *T*. *japonicum*. **1** Stromata arising from *Elaphomyces* fruiting bodies. **2** Stromata in natural habitat. **3** Vertical section of perithecia.** 4** Colony on PDA in a 6 cm Petri dish at three weeks. **5 **Ascospores articulated into part-spores.** 6 **Thickened tip of ascus. **7** Conidia. **8** Conidiogenous cell. Figure** 9**–**14** *Ophiocordyceps odonatae*. **9**, **10** Stromata produced from a dead dragonfly. **11** Sectioned stroma. **12** Medullary hyphae of stroma. **13** Conidiogenous cells. **14** Conidia. *Bars* **1**, **9** = 1 cm; **10** = 5 mm; **11** = 0.5 mm; **3** = 100 μm; **12** = 40 μm; **5**–**8**, **13**, **14** = 5 μm

Certain ophiocordycipitaceous fungi are of great medicinal potential. Salient examples include *Ophiocordyceps sinensis* (Berk.) G.H. Sung et al., the caterpillar fungus, which is a high-priced traditional Chinese medicine (Lo et al. 2013), and *Tolypocladium inflatum* W. Gams, the origin of cyclosporin (Henry 1998). *Ophiocordyceps* and *Harposporium* are the two genera of Ophiocordycipitaceae that have been reported in Taiwan (Wang et al. 1999). Most records of *Ophiocordyceps* reported in Taiwan were from ants; others were from wasps, grasshoppers, flies, stink bugs or rove beetles (Tzean et al. 1997a, and see *Key to genera of Ophiocordycipitaceae in Taiwan* herein). *Harposporium* species reported in Taiwan thus far were exclusively nematode parasites (Tzean et al. 1997b; Kuo et al. 2008).

In this study, we add to the mycobiota of Taiwan two ophiocordycipitaceous species associated with interesting hosts: *Ophiocordyceps* *odonatae* (Kobayasi) G. H. Sung et al. on dragonflies and *Tolypocladium* *japonicum* (C. G. Lloyd) Quandt et al. on subterranean ascomata of an *Elaphomyces* fungus. *Elaphomyces* and dragonflies have not been reported as hosts of ophiocordycipitaceous fungi in Taiwan.

## Methods

### Morphological observation

Specimens were air-dried and stored in the herbarium of Biodiversity Research Center, Academia Sinica, Taipei (HAST). Freehand thin sections were mounted in 5 % KOH or distilled water. Microscopic features were observed with a Leica DM2500 microscope equipped with differential interference contrast. Cultures were initiated from stromatal tissue inoculated on scratch malt extract agar (MEA) (Kenerley and Rogers 1976). Descriptions of cultures and anamorphs were made based on their morphology on potato dextrose agar (PDA) at 25 C. Fungal cultures were deposited at BCRC (the Bioresource Collection and Research Center, Hsin-chu, Taiwan).

### DNA extraction, amplification, sequencing and sequence analysis

Fungal cultures were grown in malt extract broth (2 g malt extract in 100 ml water). Total DNA was extracted from freeze-dried mycelia by automated magnetic bead operating platform Smart LabAssist (Taiwan Advanced Nanotec Inc., Taoyuan, Taiwan) with nucleic acid extraction kit TANBead^®^ Fungi DNA Auto tube (Taiwan Advanced Nanotec Inc., Taoyuan, Taiwan). PCR amplification of the internal transcribed spacers of rDNA (ITS) was described in Hsieh et al. (2009). PCR products were cleaned with DNA Advanced™ DNA Clean Up System (Viogene BioTek Corp., Sijhih, Taiwan) following the manufacturer’s protocol. Sequencing methods were as in Hsieh et al. (2009).

ITS sequence identity values were obtained by searching the nucleotide collections at NCBI by MEGABLAST, with the scores of “match/mismatch score” and “gap cost” as “1, -2″ and “linear”, respectively. For each species, the top five matches with known identities were cited in the present study.

## Results and discussion

*Tolypocladium japonicum* (C. G. Lloyd) Quandt, Kepler & Spatafora, IMA Fungus 5: 126. 2014. Figures 1–8.

≡ *Elaphocordyceps japonica* (C. G. Lloyd) G. H. Sung, J. M. Sung & Spatafora, Stud. Mycol. 57: 37 2007.

Stromata one to five emerging directly from underground ascomata of *Elaphomyces* sp., clavate, unbranched, with a rounded apex, on a cylindrical, pale gray to pale brown stipe, 3.5–7.0 cm in length, 1.8–3.0 cm long by 4–6 mm broad at fertile part, 1.5–5.0 cm long by 0.2–0.4 cm broad at stipe; surface plane, roughened by ostioles, with the fertile part tan-colored when immature, blackened when mature; interior dull yellowish green, hollow at center. Perithecia ovoid to ellipsoid, 400–500 × 180–230 μm, with papillate ostioles. Asci clavate to cylindrical, 300–350 × 9–13 μm, with a conspicuously thickened cap. Ascospores hyaline, long cylindrical, disarticulated into part-spores within asci; part spores cylindrical, with flattened ends, 10–15 × 3.0–3.4 μm.

*Cultures and anamorph.* Colonies on PDA at 25 C attaining 3 cm in 3 wk, thick, white to grayish, cottony, with brownish pigments diffusing into the media. Anamorph simplicillium-like. Conidiogenous cells solitary, long, slender, tapering toward the apex, 25–50 × 1.7–2.2 μm, about 1 μm broad at the apex. Conidia produced enteroblastically, one-celled, hyaline, smooth, elliptical to obclavate, 6.5–10.0 × 3.5–4.5 μm.

*Specimens examined.* New Taipei City, Wu-lai, Nei-dong, on *Elaphomyces* sp., 26 Apr 2014, Ju, Y.-M., Hsieh, H.-M., Ke, Y.-H., Sung, A.-N., Fan, Y.-C., Hung, S.-C. & Lin, J.-R. 103042601 (HAST); New Taipei City, Wu-lai, Nei-dong, on *Elaphomyces* sp., 20 May 2014, Ju, Y.-M., Hsieh, H.-M., Ke, Y.-H. & Chang, Y.-Y. 103052001 (HAST; culture accession number: BCRC FU30561; GenBank accession number of ITS: KT873533).

*Notes.* Records of *T. japonicam* are fairly rare even in Japan (Kobayasi and Shimizu 1960) and appear sporadic in other countries, including Austria (Mains 1957; Kobayasi and Shimizu 1960) and China (Liang et al. 2003). Reflected in studies of recent years is the paucity of available material of this fungus: the DNA sequences deposited at GenBank resulting from two Japanese sources only, a specimen OSC 110991 and a culture IFO 9647. Among the three ITS sequences of *T*. *japonicum* cited below, AB027366 and EU039882 are from IFO 9647, while JN049824 is from OSC 110991.

The Taiwan collections fit well the descriptions of *T*. *japonicum* given by Kobayasi and Shimizu (1960). Besides *T.* *japonicum*, four other *Tolypocladium* species also parasitize *Elaphomyces* fruiting bodies and produce clavate stromata, including *T. ophioglossoides* (Ehrhart) Quandt et al., *T. jezoense* (S. Imai) Quandt et al., *T. tenuisporum* (Mains) Quandt et al., and *T. szemaoense* (M. Zang) Quandt et al. (Mains 1957; Kobayasi and Shimizu 1960.; Zang 2001). *Tolypocladium ophioglossoides* and *T. jezoense* differ from *T*. *japonicum* in forming a rhizomorphous structure on the stromatal base, and *T. tenuisporum* and *T. szemaoense* differ in having smaller part-spores.

A MEGABLAST query in GenBank using the ITS sequence obtained from the specimen 103052001 resulted in the following five top matches: *Elaphocordyceps japonica* (AB027366, query coverage = 100 %, identities = 596/601 [99 %], gaps = 0/601 [0 %]); *Elaphocordyceps japonica* (EU039882, query coverage = 100 %, identities = 596/602 [99 %], gaps = 1/602 [0 %]); *Elaphocordyceps japonica* (JN049824, query coverage = 93 %, identities = 393/404 [97 %], gaps = 2/404 [0 %]); *Cordyceps guangdongensis* (EU039881, query coverage = 100 %, identities = 569/608 [94 %], gaps = 28/608 [4 %]); and *Tolypocladium inflatum* (JF796050, query coverage = 95 %, identities = 511/578 [88 %], gaps = 32/578 [5 %]). The query result reinforces the Taiwan material being *T.* *japonicum.*

This is the first report describing cultures and anamorph of *T. japonicum*. The conidiogenous cells of *T*. *japonicum* are long, slender, lacking an inflated base. *Tolypocladium*, typified by *T*. *inflatum* W. Gams, was originally characterized by conidiogenous cells with an inflated base and a narrow neck. However, the genus is currently circumscribed on the basis of molecular phylogeny rather than morphology (Quandt et al. 2014).

*Ophiocordyceps odonatae* (Kobayasi) G. H. Sung, J. M. Sung, Hywel-Jones & Spatafora, Stud. Mycol. 57: 45. 2007. Figures 9–14.

≡ *Cordyceps odonatae* Kobayasi, Bull. Nat. Sci. Mus. Tokyo, Ser. B, 7: 6. 1981.

= *Hymenostilbe odonatae* Kobayasi, Sci. Rep. Tokyo Bunrika Daig., Sect. B 5: 223.1941.

Teleomorph not produced. Anamorph synnematous. Synnemata gregarious, protruding from abdominal and thoracic joints of dragonflies, pale yellow to pale orange, clavate, abruptly rounded on top, stipitate, curved towards the front of dragonflies, 3–6 mm long, 1–2 mm diam; interior white, consisting of densely interwoven hyphae of 3.5–4.5 μm in width. Sporulating region distributed mainly on convex side of synnemata, forming a more light-colored and slightly fluffy region. Conidiogenous cells hyaline, clavate, apiculate on tip, 13–21 × 2.5–3.2 μm, warted. Conidia produced holoblastically in sympodial sequence, one-celled, hyaline, cylindrical to fusiform, 10–12 × 2.5–3.5 μm.

*Cultures and anamorph.* Colonies on PDA at 25 C attaining 1 cm in 3 wk, white, overlain with short dense aerial hyphae, diffuse at margins. Sporulation absent.

*Specimens examined.* I-lan County, Da-jiao-si Experimental Forest, on *Planaeschna* sp. (Odonata: Aeshnidae), 27 Apr 2013, Ju, Y.-M. & Hsieh, H.-M. 102042701 (HAST; culture accession number: BCRC FU30560; GenBank accession number of ITS: KT873534). New Taipei City, Wu-lai, on *Planaeschna* sp. (Odonata: Aeshnidae), 14 Jul 2013, Ke, Y.-H. 102071404 (HAST).

*Notes.* *Ophiocordyceps odonatae* is the only species in the genus known to parasitize dragonflies. The two studied Taiwan collections were made from the dragonfly genus *Planaeschna*, from which the type specimen of *Hymenostilbe odonatae* Kobayasi was also collected (Kobayasi 1941, 1981). Only the anamorph was present in the Taiwan collections. It should be noted that the basionym of *O*. *odonatae* is the teleomorph-typified binomial *Cordyceps* *odonatae* Kobayasi, which is predated by the anamorph-typified binomial *Hymenostilbe* *odonatae*. Recombining the epithet of *H*. *odonatae* with *Ophiocordyceps* would result in an illegitimate later homonym of *O*. *odonatae* (Kobayasi) G. H. Sung et al.

Conidiogenous cells and conidia in Taiwan collections are slightly larger than those documented in the protologue of *H.* *odonatae* (Kobayasi 1941). Also, unlike the more or less curved conidia described in Kobayasi (1941), conidia in Taiwan collections are not curved or only slightly curved. Modes of conidiogenesis were considered an important character in separating *Hymenostilbe* from *Akanthomyces* Lebert by Samson and Evans (1975), with the former producing conidia holoblastically in sympodial sequence and the latter enteroblastically. Samson and Evans (1975) related *H.* *odonatae* to *Akanthomyces* with reference to the description in Kobayasi (1941). Our study clearly shows that the anamorph of *O*. *odonatae* produces conidia holoblastically in sympodial sequence and can be accommodated in *Hymenostilbe*.

A MEGABLAST query in GenBank using the ITS sequence obtained from the specimen 102042701 resulted in the following five top matches: *Hymenostilbe odonatae* (AB104725, query coverage = 100 %, identities = 576/581 [99 %], gaps = 1/581 [0 %]); *Ophiocordyceps forquignonii* (HQ662164, coverage = 33 %, identity = 187/198 [94 %], gaps = 3/198 [1 %]); *Cordyceps forquignonii* (AJ786562, coverage = 33 %, identity = 187/198 [94 %], gaps = 3/198 [1 %]); uncultured *Volutella* (HM136667, coverage = 42 %, identity = 175/187 [94 %], gaps = 3/187 [1 %]); and *Phialophora* sp. (AJ010039, coverage = 35 %, identity = 187/205 [91 %], gaps = 4/205 [1 %]). The query result shows a good match between our materials and *O.* *odonatae*.

In addition to our report in Taiwan, *O. odonatae* had previously been recorded in Japan (Kobayasi 1941), northeastern and southern China (Song et al. 2006; Yang et al. 2004), and New Guinea (Kobayasi 1981), being widely distributed across climate zones from temperate regions to the tropics. Future collections may reveal that *O. odonatae* is distributed mainly along the Western Pacific Region.

## Key to species of Ophiocordycipitaceae in Taiwan

- 1. Associated with nematodes...2 (*Harposporium*)
  [The *Harposporium* species found in Taiwan were anamorphic only.]
- 1. Associated with arthropods or hypogeous fungi...4
-
  - 2. Conidia straight, cylindrical, swelling at two ends, 5–6 μm long...*H. bysmatosporum* Drechsler (Kuo et al. 2008).
  - 2. Conidia curved or coiled...3
- 3. Conidia falcate, pointed at both ends, 7–13 μm long...*H. anguillulae* Lohde (Tzean et al. 1997b, Kuo et al. 2008).
- 3. Conidia coiled, 8–10 μm long straightly between two ends...*H. leptospira* Drechsler (Kuo et al. 2008).
  - 4. Stromata clavate or capitate, associated with hypogeous fungi of the genus *Elaphomyces*; anamorph simplicillium-like...*Tolypocladium japonicum* (herein).
  - 4. Stromata clavate or capitate, associated with arthropods; anamorphs hirsutella-like or hymenostilbe-like...5 (*Ophiocordyceps*).
- 5. Associated with Endopterygota insects (ants, wasps, beetles, flies, dragonflies)...6
- 5. Associated with Exopterygota insects (leafhoppers, stink bugs)...13
  - 6. Associated with Hymenoptera insects (ants, wasps)...7
  - 6. Associated with Endopterygota insects other than Hymenoptera...11
- 7. Associated with wasps...*O. humbertii* (C. P. Robin) G. H. Sung et al. (Tzean et al. 1997a).
- 7. Associated with ants...8
  - 8. Ascospores not breaking into part-spores; conidiogenous cells clustering at synnematal apex, echinulate; conidia ellipsoid to cylindrical, 8–11 μm long...*O. unilateralis* (Tul. & C. Tul.) Petch (Tzean et al. 1997a).
  - 8. Ascospores breaking into part-spores...9
- 9. Part-spores fusoid to ellipsoid, not distinctly truncate, without frilled ends...*O. irangiensis* (Moureau) G. H. Sung et al. (Tzean et al. 1997a).
- 9. Part-spores barrel-shape, distinctly truncate, with frilled ends...10
  - 10. Stromata with a short, rigid stipe shorter than five times of the fertile part...*O. pseudolloydii* (H. C. Evans & Samson) G. H. Sung et al. (Tzean et al. 1997a).
  - 10. Stromata with a long, slender stipe longer than five times of the fertile part...*O. myrmecophila* (Ces.) G. H. Sung et al. (Chen 1978; Tzean et al. 1997a).
- 11. Associated with rove beetles (Coleoptera)...*O. kniphofioides* (H. C. Evans & Samson) G. H. Sung et al. (Tzean et al. 1997a)
  [Evans and Samson (1982) described *O. kniphofioides* and its anamorph *Hirsutella* *stilbelliformis* var. *stilbelliformis* from ants in Brazil. *Hirsutella* *stilbelliformis* var. *stilbelliformis* identified by Tzean et al. (1997a) is associated with rove beetles.]
- 11. Associated with insects other than beetles (Coleoptera)...12
  - 12. Associated with dragonflies...*O. odonatae* (herein).
  - 12. Associated with flies...*O. dipterigena* (Berk. & Broome) G. H. Sung et al. (Chen 1978; Tzean et al. 1997a).
- 13. Associated with stink bugs...*O. nutans* (Pat.) G. H. Sung et al. (Chen 1978; Tzean et al. 1997a).
- 13. Associated with leafhoppers...“*Hirsutella*” *versicolor* Petch (Tzean et al. 1997a)
  [This fungus may eventually be placed in *Ophiocordyceps*. Its teleomorph has not been found thus far.]
